# Soluble mucosal addressin cell adhesion molecule 1 is a biomarker for pediatric ulcerative colitis

**DOI:** 10.1186/s40348-026-00221-8

**Published:** 2026-02-24

**Authors:** Moritz Muschaweck, Christian Gutbier, Gernot Sellge, Angeliki Pappa, Tobias Wenzl, Karim Hamesch, Norbert Wagner, Angela Schippers

**Affiliations:** 1 Department of Pediatrics, RWTH Aachen University, Aachen, Germany; 2 Department of Medicine III, RWTH Aachen University, Aachen, Germany; 3 Department for Internal Medicine II, Klinikum Bremen Mitte, Bremen, Germany; 4 Department of Pediatrics, RWTH University Hospital Aachen, Pauwelsstraße 30, D- 52074 Aachen, Germany

**Keywords:** Inflammatory bowel disease, Lymphocyte migration, Bioassay

## Abstract

**Background:**

Enhanced expression of mucosal addressin cell adhesion molecule 1 (MAdCAM-1) on high endothelial venules facilitates lymphocyte migration to sites of intestinal inflammation in inflammatory bowel disease (IBD). This process may be accompanied by increased shedding of soluble MAdCAM-1 (sMAdCAM-1) from endothelial cells. Hence, circulating sMAdCAM-1 may serve as a biomarker of intestinal inflammation in IBD.

**Results:**

This single-center study enrolled pediatric (*n* = 32) and adult (*n* = 73) patients with IBD, as well as healthy controls (*n* = 56). Serum samples were collected from all participants, and sMAdCAM-1 concentrations were quantified using an ELISA assay. In healthy individuals, sMAdCAM-1 levels were highest in early childhood, declined during adolescence, and were lowest in adulthood. Concentrations of sMAdCAM-1 did not correlate with gut inflammation in pediatric or adult patients with Crohn’s disease (CD). In contrast, significantly increased sMAdCAM-1 levels were observed in adults with severe ulcerative colitis (UC). Moreover, sMAdCAM-1 levels distinguished healthy children from pediatric UC patients.

**Conclusions:**

These findings provide new insights into sMAdCAM-1 as a marker of gut homeostasis and inflammation. Shedding of sMAdCAM-1 decreases during adolescence, and circulating sMAdCAM-1 levels could serve as a biomarker for ulcerative colitis in both pediatric and adult patients, particularly in severe disease.

## Background

Immigration of leukocytes into the intestinal mucosa represents the decisive precondition for initiation and perpetuation of inflammatory bowel disease (IBD) [1–3]. Chemokines and adhesion molecules regulate a multistep cascade of lymphocyte rolling, homing and endothelial transmigration [4]. Firm adhesion of lymphocytes by interaction of α4β7 integrin with mucosal addressin cell adhesion molecule 1 (MAdCAM-1) expressed on high endothelial venules (HEV) initializes the transmigration of T and B cells into gut-associated lymphatic tissue (GALT) [5].

Considerable enhancement of MAdCAM-1 expression on HEV accompanied by increased influx of leukocytes is a typical feature of both IBD patients and murine colitis models [6–9]. Anti-adhesion molecule therapies such as vedolizumab, a humanized monoclonal antibody against α4β7 integrin that blocks lymphocyte transmigration, provide new options for IBD treatment [10]. Randomized, controlled trials have confirmed the efficacy and safety of vedolizumab for both ulcerative colitis (UC) and Crohn´s disease (CD) treatment [11–13].

Our understanding of IBD etiology is still incomplete but a complex interaction of genetic and environmental factors (e.g., food, hygiene or antibiotics) appears to be involved [14, 15]. Moreover, the global incidence of IBD is still on the rise, particularly in children and adolescents [16, 17]. Gut immunity represents the central element in IBD pathogenesis and its primary therapeutic target. An imbalanced immune reaction towards commensal microbiota or food antigens may be involved in IBD pathogenesis [18–21]. Thus, infant maturation of adaptive immune homeostasis towards commensals and food antigens exhibits basic prerequisites for gut health in later adulthood.

The aim of our study was to verify the capability of soluble MAdCAM-1 (sMAdcAM-1) to serve as a biomarker for pediatric and adult IBD. Gene transcription of *MAdCAM-1* is upregulated by pro-inflammatory cytokines like tumor necrosis factor α (TNFα) [2, 22, 23]. IBD patients and murine colitis models both exhibit enhanced MAdCAM-1 expression on HEV [2, 24, 25]. In fact, cell adhesion molecules (CAM) like vascular cell adhesion molecule 1 (VCAM-1) or intercellular adhesion molecule-1 (ICAM-1) constitutively shed from endothelial cells at inflamed sites and circulate in bloodstream as soluble CAMs. Soluble CAMs could therefore serve as serologic biomarkers in several acute and chronic diseases [26, 27] while elevated concentrations of sMAdCAM-1 could potentially mirror the increase of MAdCAM-1 expression in the intestine of IBD patients.

## Materials and methods

### Patients

**Fig. 1:**
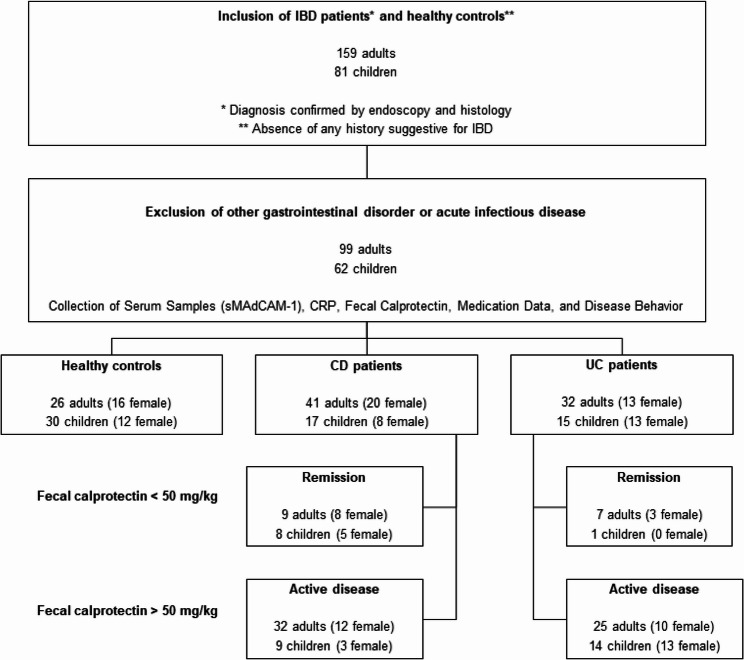
Enrollment of adult and pediatric study populations for sMAdCAM-1 assessment. Between October 2014 and February 2016, pediatric and adult IBD patients, along with healthy control participants, were recruited through the Department of Pediatrics and the Department of Medicine III at RWTH Aachen University Hospital. After applying inclusion and exclusion criteria, 56 healthy controls (30 pediatric), 58 CD patients (17 pediatric), and 47 UC patients (15 pediatric) were assessed for sMAdCAM-1 measurement. IBD patients were categorized based on fecal calprotectin levels into remission (< 50 mg/kg) or active disease (> 50 mg/kg)

From October 2014 to February 2016, pediatric and adult IBD patients, along with healthy control participants, were recruited through the Department of Pediatrics and the Department of Medicine III at RWTH Aachen University Hospital, North Rhine-Westphalia, Germany (Fig. 1). Inclusion criteria for IBD patients were a confirmed diagnosis of UC or CD, verified by both endoscopic and histological examination. Inclusion criteria for healthy controls were the absence of any history suggestive of IBD. Exclusion criteria for all participants were evidence of other gastrointestinal or infectious disorders based on clinical assessment at the time of sample collection. Following application of the inclusion and exclusion criteria, 56 healthy controls (including 30 pediatric subjects), 58 CD patients (17 pediatric), and 47 UC patients (15 pediatric) were enrolled. All IBD patients were receiving maintenance therapy and no newly diagnosed IBD patients were included.

For both pediatric and adult IBD patients, fecal calprotectin and CRP levels were routinely determined while serum samples were collected at the same visit for subsequent sMAdCAM-1 analysis. According to fecal calprotectin levels, IBD patients were divided into remission (< 50 mg/kg) or active (> 50 mg/kg) disease (Fig. 1). The cut-off value of 50 mg/kg was selected based on evidence from a published meta-analysis demonstrating that this threshold provides high sensitivity for detecting active IBD 28. Moreover, IBD patients with active disease were classified as mild/moderate (< 500 mg/kg) or severe (> 500 mg/kg) based on fecal calprotectin levels. Cohort characteristics are summarized in Tables 1 and 2.

**Table 1:** Characteristics of the adult cohorts

Healthy controls, *n* [%]	**26**	**[26.3%]**
Female, *n* [%]	16	[61.5%]
Age, *y*, median [IQR_25-75_]	50.8	[24.7 - 58.5]
Age 18-40 y, *n* [%]	12	[46.2%]
Age > 40 y, *n* [%]	14	[53.8%]
Age 18-40 y, *y*, median [IQR_25-75_]	26.2	[24.7 - 28.6]
Age > 40 y, *y*, median [IQR_25-75_]	59.5	[53.8 - 63.5]
IBD patients, *n* [%]	**73**	**[73.7%]**
Female, *n* [%]	33	[45.2%]
Age, *y*, median [IQR_25-75_]	43.9	[33.2 - 54.1]
Crohn, *n* [%]	**41**	**[41.4%]**
Female, *n* [%]	20	[48.8%]
Age, *y*, median [IQR_25-75_]	41.2	[30.7 - 49.8]
Age 18-40 y, *n* [%]	19	[46.3%]
Age > 40 y, *n* [%]	22	[53.7%]
Age 18-40 y, *y*, median [IQR_25-75_]	31.3	[25.3 - 35.1]
Age > 40 y, *y*, median [IQR_25-75_]	48.2	[40.1 - 57.5]
Remission (Calprotectin <50 mg/kg), *n* [%]	9	[22.0%]
Active (Calprotectin >50 mg/kg), n [%]	32	[78.0%]
Mild/Moderate (Calprotectin 50-500 mg/kg), *n* [%]	20	[48.8%]
Severe (Calprotectin >500 mg/kg), *n *[%]	12	[29.2%]
Calprotectin, *mg/kg*, median [IQR_25-75_]	182	[65 - 503]
Remission (Calprotectin <50 mg/kg)	<50	
Active (Calprotectin >50 mg/kg)	234	[145 - 539]
Mild/Moderate (Calprotectin 50-500 mg/kg)	151	[123 - 227]
Severe (Calprotectin >500 mg/kg)	704	[513 - 1,600]
Behavior, *n *[%]		
non-stricturing, non-penatrating [B1]	20	[48.8%]
stricturing [B2]	15	[36.6%]
penetrating [B3]	6	[14.6%]
Location, *n *[%]		
Terminal ileum	9	[22.0%]
Ileocolonic	22	[53.7%]
Colonic	5	[12.2%]
Undefined	5	[12.2%]
Ulcerative colitis, *n* [%]	**32**	**[32.3%]**
Female, *n* [%]	13	[40.6%]
Age, median [IQR_25-75_]	43.9	[34.5 - 58.4]
Age 18-40 y, *n* [%]	13	[40.6%]
Age > 40 y, *n* [%]	19	[59.4%]
Age 18-40 y, *y*, median [IQR_25-75_]	30.9	[24.4 - 34.8]
Age > 40 y, *y*, median [IQR_25-75_]	53.7	[44.6 - 65.1]
Remission (Calprotectin <50 mg/kg), *n* [%]	7	[21.9%]
Active	25	[78.1%]
Mild/Moderate (Calprotectin 50-500 mg/kg), *n* [%]	14	[43.8%]
Severe (Calprotectin >500 mg/kg), *n *[%]	11	[34.4%]
Calprotectin, *mg/kg*, median [IQR_25-75_]	208.4	[42.73 - 1,468]
Remission (Calprotectin <50 mg/kg)	<50	
Active (Calprotectin >50 mg/kg)	395	[145 - 1,600]
Mild/Moderate (Calprotectin 50-500 mg/kg)	145	[106 - 240]
Severe (Calprotectin >500 mg/kg)	1,600	[1,067 - 1,600]
Location, *n *[%]		
Left-sided	18	[56.3%]
Pancolitis	12	[37.5%]
Undefined	2	[6.3%]

**Table 2:** Characteristics of the pediatric cohorts

Healthy controls, *n* [%]	**30**	**[48.4%]**
Female, *n* [%]	12	[40.0%]
Age, *y*, median [IQR_25-75_]	11.2	[5.1 - 14.3]
Age < 8, *n* [%]	12	[40.0%]
Age > 8, *n* [%]	18	[60.0%]
Age 8-14 y, *n* [%]	8	[26.7%]
Age 14-18 y, *n* [%]	10	[33.3%]
Age < 8, *y*, median [IQR_25-75_]	4.1	[3.4 - 6.3]
Age > 8, *y*, median [IQR_25-75_]	14.1	[11.7 - 15.7]
Age 8-14 y, *y*, median [IQR_25-75_]	11.6	[10.8 - 11.8]
Age 14-18 y, *y*, median [IQR_25-75_]	15.3	[14.2 - 16.8]
CRP, *mg/l*, median [IQR_25-75_]	0.5	[0.3 - 4.4]
IBD patients, *n* [%]	**32**	**[51.6%]**
Female, *n* [%]	21	[65.6%]
Age, *y*, median [IQR_25-75_]	14.8	[13.7 - 17.1]
Crohn, *n* [%]	**17**	**[27.4%]**
Female, *n* [%]	8	[47.1%]
Age, *y*, median [IQR_25-75_]	17.1	[14.4 - 17.6]
Age > 8, *n* [%]	17	[100%]
Age 8-14 y *n* [%]	3	[17.6%]
Age 14-18 y, *n* [%]	14	[82.4%]
Age 8-14 y, *y*, median [IQR_25-75_]	10.8	[9.5 - 13.9]
Age 14-18 y, *y*, median [IQR_25-75_]	17.2	[15.3 - 17.6]
Remission (Calprotectin <50 mg/kg), *n* [%]	8	[47.1%]
Active (Calprotectin >50 mg/kg), n [%]	9	[52.9%]
Mild/Moderate (Calprotectin 50-500 mg/kg), *n* [%]	3	[17.6%]
Severe (Calprotectin >500 mg/kg), n [%]	6	[35.3%]
CRP, *mg/l*, median [IQR_25-75_]	1.2	[0.4 - 26.9]
Remission (Calprotectin <50 mg/kg)	0.6	[0.3 - 7.3]
Active (Calprotectin >50 mg/kg)	2.4	[0.7 - 47.8]
Mild/Moderate (Calprotectin 50-500 mg/kg)	0.7	[0.3 - 1.0]
Severe (Calprotectin >500 mg/kg)	2.4	[0.4 - 45.3]
Calprotectin, *mg/kg*, median [IQR_25-75_]	227	[39 - 1,600]
Remission(Calprotectin <50 mg/kg)	<50	
Active (Calprotectin >50 mg/kg)	1,600	[449 - 1,600]
Mild/Moderate (Calprotectin 50-500 mg/kg)	151	[75 - 227]
Severe (Calprotectin >500 mg/kg)	1,600	[900 - 1,600]
Localization		
Terminal Ileum	3	[17.6%]
Ileocolonic	6	[35.3%]
Colonic	8	[47.1%]
Medication		
Azathioprine	6	[35.3%]
Azathioprine + Corticosteroid	1	[6.7%]
TNFa antagonist	3	[17.6%]
TNFa antagonist + Azathioprine	5	[29.4%]
a4b7 antagonist + Azathioprine	2	[11.8%]
Ulcerative colitis, *n* [%]	**15**	**[24.2%]**
Female, *n* [%]	13	[86.7%]
Age, *y*, median [IQR_25-75_]	13.8	[10.4 - 15.7]
Age > 8, *n* [%]	15	[100%]
Age 8 -14 y *n* [%]	6	[40%]
Age 14-18 y, *n* [%]	9	[60%]
Age 8-14 y, *y*, median [IQR_25-75_]	13.7	[10.3 - 13.7]
Age 14-18 y, *y*, median [IQR_25-75_]	15.3	[14.1 - 16.0]
Remission (Calprotectin <50 mg/kg), *n* [%]	1	[6.7%]
Active (Calprotectin >50mg/kg), *n* [%]	14	[93.3%]
Mild/Moderate (Calprotectin 50-500 mg/kg)	7	[46.7%]
Severe (Calprotectin >500 mg/kg)	7	[46.7%]
CRP, *mg/l*, median [IQR_25-75_]	1.3	[0.4 - 2.1]
Remission (Calprotectin <50 mg/kg)	0.3	
Active (Calprotectin >50 mg/kg)	1.5	[0.48 - 2.2]
Mild/Moderate (Calprotectin 50-500 mg/kg)	0.4	[0.3 - 3.5]
Severe (Calprotectin >500 mg/kg)	2.1	[1.1 - 2.3]
Calprotectin, *mg/kg*, median [IQR_25-75_]	506	[39 - 1,600]
Remission(Calprotectin <50 mg/kg)	<50	
Active (Calprotectin >50 mg/kg)	747	[350 - 1,600]
Mild/Moderate (Calprotectin 50-500 mg/kg)	242	[119 - 431]
Severe (Calprotectin >500 mg/kg)	1,155	[747 - 1,600]
Localization		
Pancolitis	15	[100%]
Medication		
Azathioprine	5	[33.3%]
TNFa antagonist + Azathioprine	7	[46.7%]
a4b7 antagonist + Azathioprine	3	[20.0%]

All patients and healthy controls – or their parents or legal guardians, in the case of minors – provided written informed consent in accordance with the protocol approved by the Independent Ethics Committee of RWTH Aachen University Hospital (approval numbers EK 049/12 and EK 112/15).

### Detection of sMAdCAM-1 concentrations

Immediately after collection, blood samples were centrifuged and the supernatants stored at − 20 °C until subsequent analysis. sMAdCAM-1 concentrations were detected using the human sMAdCAM-1 ELISA assay (Hycult Biotech Inc) according to the manufacturer’s instructions 29. The sMAdCAM-1 ELISA assay is a ready-to-use solid-phase enzyme-linked immunosorbent assay based on the sandwich principle for the in vitro quantitative determination of human sMAdCAM-1 in serum samples. Detected concentration levels range from 0.41 to 100 ng/ml. Each sample was analyzed in duplicate, and each assay included internal controls and a standard calibration series to ensure accuracy and reliability of the measurements.

### Statistical analysis

The primary objective of our study was to analyze the biomarker capabilities of sMAdCAM-1 for pediatric and adult IBD patients. Differences in sMAdCAM-1 concentrations between healthy subjects and IBD patients were determined by Student´s t-test or one-way analysis of variance (ANOVA) and Tukey post-hoc test (*p* < 0.05). Receiver operating characteristics (ROC) curves were plotted to illustrate the diagnostic ability of sMAdCAM-1 determination for pediatric and adult cohorts. Areas under the ROC curves (AUC) were then calculated.

Secondly, we analyzed the dependence of sMAdCAM-1 expression on age, using Spearman´s rank correlation as a nonparametric measure of rank correlation. A ρ-value of -1 or + 1 signifies perfect positive or negative rank correlation, respectively. Student´s t test determined the significance of the rank correlation.

Thirdly, we used descriptive statistics for characterization of our different cohorts by calculating median values and interquartile ranges (IQR_25−75_).

For all comparative analyses of pediatric IBD patients, we used subgroups of age-matched controls older than 8 years.

## Results

### Age-related decrease of sMAdCAM-1 concentrations throughout healthy infancy, adolescence, and adulthood

Over the period from October 2014 to February 2016 serum samples from cohorts of both pediatric (*n* = 30) and adult healthy individuals (*n* = 26) were analyzed, and sMAdCAM-1 concentrations determined by sMAdCAM-1 ELISA assay (Hycult Biotech Inc). Characteristics of the adult and pediatric cohorts are shown in Tables 1 and 2, respectively. Within the pediatric cohort 40.0% were female and the median age was 11.2 years (IQR_25−75_ 5.1–14.3) with 18 subjects older than 8 years (60.0%). 61.5% of the adult subjects were females, and the median age was 50.8 years (IQR_25−75_ 24.7–58.5).

**Fig. 2:**
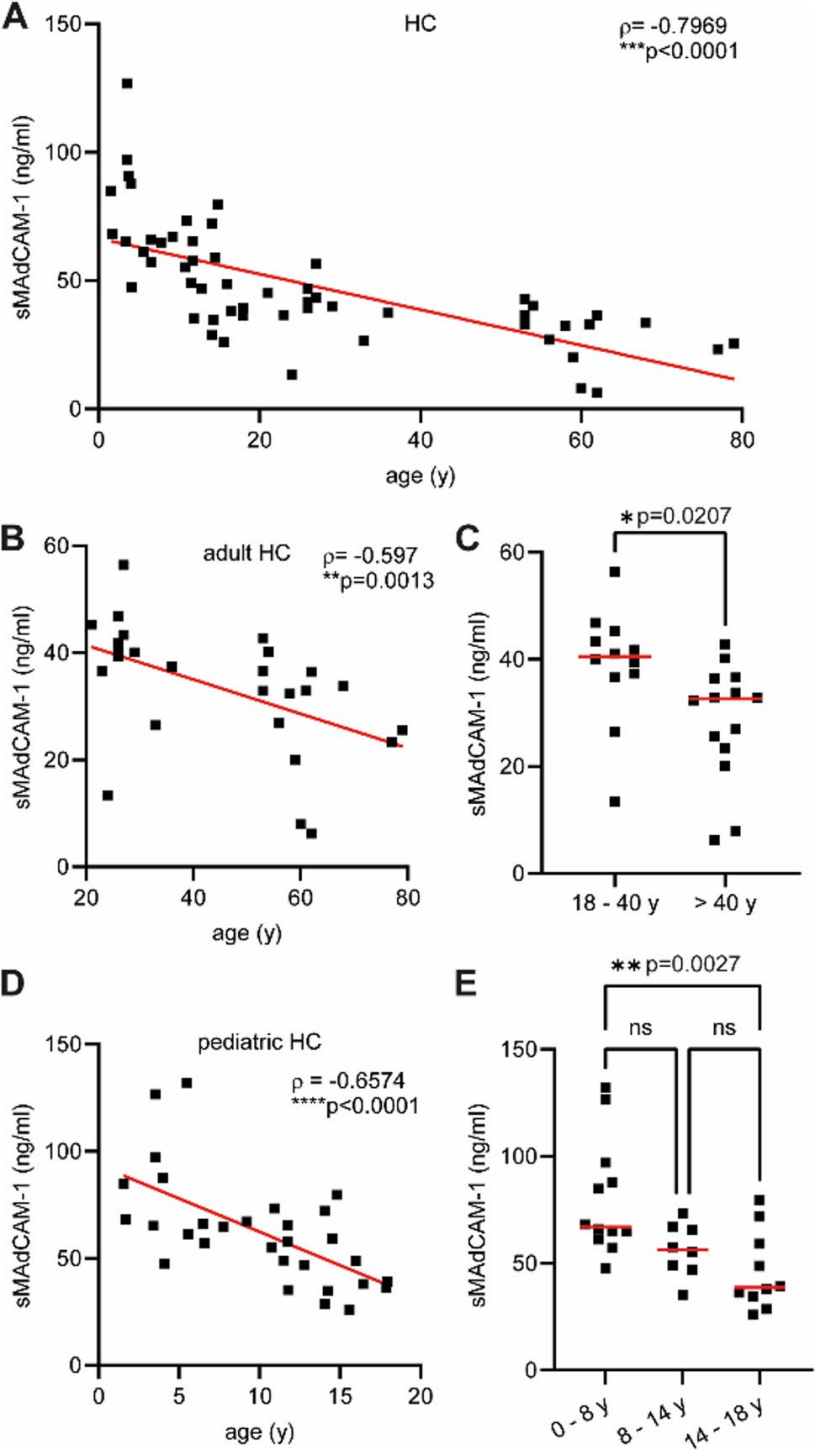
Age-related decrease of sMAdCAM-1 concentrations throughout infancy, adolescence and adulthood. sMAdCAM-1 concentrations were determined by sMAdCAM-1 detection assay in serum samples from 30 pediatric and 26 adult individuals without intestinal inflammation (healthy controls; HC). Spearman rank correlation between sMAdCAM-1 concentrations [ng/ml] and age [y] were calculated for all healthy subjects (**A**) and for adults (**B**) or children (**D**) separately. Dots represent individual samples while red lines represent linear regression. Negative correlations were observed for all three comparisons. Age-related decrease of sMAdCAM-1 was significant for the comparison of adults subclassified by age 18-40 (n=12) and age >40 (n=14) years (**C**). sMAdCAM-1 concentrations from subpopulations of children younger (n=12) or older than 8 years (n=18) differed remarkably (**E**). Red horizontal lines represent the mean. *p* values for Spearman's rank correlations and p values of Student's t-tests or Tukey post-hoc tests are indicated

Initially, we studied the interrelation of age development and sMAdCAM-1 levels in healthy individuals by calculation of Spearman´s rank correlations. Our study revealed an inverse correlation between sMAdCAM-1 concentration and age. sMAdCAM-1 levels consistently decreased throughout infancy, childhood, adolescence and adulthood (ρ=-0.7969; ****p < 0.0001) (Fig. 2A). This observation was still apparent when looking at the adult cohort separately (ρ=-0.597; **p = 0.0013) (Fig. 2B). Notable differences in sMAdCAM-1 levels were detected when healthy individuals from different age groups were compared (18-40y (*n* = 12): 39.00 ± 10.69 ng/ml; >40y (*n* = 14): 28.42 ± 11.00 ng/ml; *p = 0.0207) (Fig. 2C). Strikingly, this observed inverse correlation between sMAdCAM-1 concentration levels and age was even more drastic in our pediatric cohort (ρ=-0.6574; ****p < 0.0001). (Fig. 2D). Pediatric subjects from different age groups displayed fundamentally different sMAdCAM-1 levels (0-8y (*n* = 12): 79.70 ± 26.65 ng/ml; 8-14y (*n* = 8): 56.24 ± 12.42 ng/ml; 14-18y (*n* = 10): 46.25 ± 18.32 ng/ml; **p = 0.0027) (Fig. 2E).

**Fig. 3:**
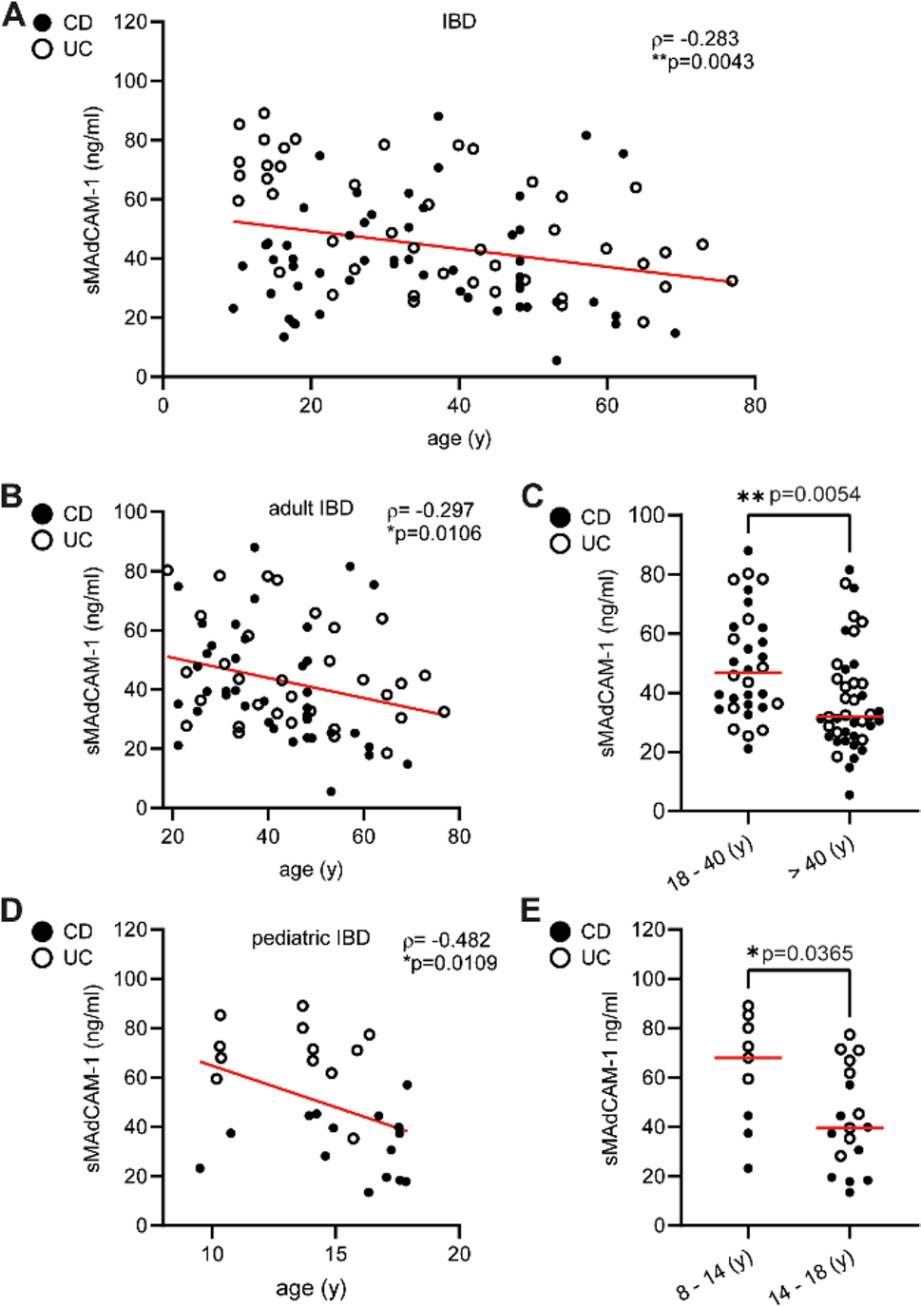
Age-related decrease of sMAdCAM-1 concentrations throughout infancy, adolescence and adulthood. sMAdCAM-1 concentrations were determined by sMAdCAM-1 detection assay in serum samples from 32 pediatric and 73 adult patients with inflammatory bowel disease (IBD). Spearman rank correlation between sMAdCAM-1 concentrations [ng/ml] and age [y] were calculated for all IBD patients (**A**) and for adults (**B**) or children (**D**) separately. Dots represent individual samples while red lines represent linear regression. Solid symbols denote Crohn’s disease (CD), while open symbols denote ulcerative colitis (UC). Negative correlations were observed for all three comparisons. Age-related decrease of sMAdCAM-1 was significant for the comparison of adults subclassified by age 18–40 years (n = 32) and age > 40 years (n = 41) (**C**). Pediatric IBD patients were subdivided into two age groups (8–14 years; n = 9, and 14–18 years; n = 18). Serum sMAdCAM-1 concentrations differed markedly among these groups (**E**). Red horizontal lines represent the mean. ρ values for Spearman´s rank correlations and p values of Student´s t-tests are indicated

Next, we evaluated whether the inverse correlation between sMAdCAM-1 shedding and age observed in healthy individuals also applied to adult and pediatric IBD patients. We collected serum samples from 73 adult IBD patients (median age 43.9 years; IQR_25−75_ 33.2–54.1) and 32 pediatric IBD patients (median age 14.8 years; IQR_25−75_ 13.7–17.1), including patients both in remission and with active disease, and quantified sMAdCAM-1 concentrations. Consistent with our findings in healthy individuals, sMAdCAM-1 serum levels declined with increasing age in IBD patients. However, the strength of this correlation was markedly reduced compared with healthy controls (ρ=-0.283; **p = 0.0043) (Fig. 3A). When adult and pediatric IBD cohorts were analyzed separately, a significant inverse correlation between age and sMAdCAM-1 levels was still observed in both groups (adult cohort: ρ=-0.297; *p = 0.0106; pediatric cohort: ρ=-0.482; *p = 0.0109) (Fig. 3B and D). Stratification of adult and pediatric IBD patients into age-defined subgroups revealed significant differences in sMAdCAM-1 serum concentrations accordingly. Adult patients younger than 40 years (*n* = 32; 49.57 ± 17.98 ng/ml) exhibited higher sMAdCAM-1 levels than patients older than 40 years (*n* = 41; 37.52 ± 17.65 ng/ml; **p = 0.0054) (Fig. 3E). Similarly, pediatric patients aged 8–14 years (*n* = 9; 62.19 ± 22.83 ng/ml) showed significantly higher sMAdCAM-1 levels compared with those aged 14–18 years (*n* = 18; 43.08 ± 20.37 ng/ml; *p = 0.0365) (Fig. 3E).

### sMAdCAM-1 levels are increased in adults with severe ulcerative colitis

To compare sMAdCAM-1 concentrations of healthy individuals with IBD patients, we took serum samples from CD (*n* = 41) and UC (*n* = 32) adult patients. Characteristics of both IBD entities are reported in Table 1. Median ages of CD (41.2 years; IQR_25−75_ 30.7–49.8) and UC patients (43.9 years; IQR_25−75_ 34.5–58.4) were comparable. At the time-point of sample acquisition, 22.0% of the CD patients (*n* = 9) were in remission, whereas the majority (*n* = 32; 78.0%) exhibited active disease. Among patients with active CD, 12 individuals (29.3%) were classified as having severe disease. Within the UC cohort, 21.9% of patients (*n* = 7) were in remission, while 78.1% (*n* = 25) exhibited active disease, including 11 patients (34.4%) classified as having a severe course of disease. Patients were further ranked by disease behavior or endoscopic localization of inflammation as detailed in Table 1. Table 3 summarizes sMAdCAM-1 concentrations across all cohorts.

**Table 3:** Serum sMAdCAM-1 levels (ng/ml) of adult cohorts (healthy controls and IBD patients)

	**Mean**	**SD**	**Median**	**IQR**_25-75_
Healthy controls	**33.3**	**±11.92**	**36.52**	**26.26 - 41.22**
18-40 years	39	±10.69	32.6	36.78 - 44.81
>40 years	28.42	±11.00	32.6	22.53 - 36.49
Adults IBD patients	**42.8**	**±18.67**	**38.21**	**28.82 - 55.96**
18-40 years	49.57	±17.98	46.86	35.32 - 62.23
>40 years	37.52	±17.65	31.92	25.30 - 46.36
CD	**41.03**	**±19.32**	**36**	**26.06 - 53.45**
Remission	43.63	±9.81	47.54	34.43 - 50.87
Active	41.08	±21.32	36.52	26.26 - 41.22
18-40 years	51.59	±17.76	45.07	37.61 - 64.17
>40 years	33.91	±18.79	29.4	23.29 - 41.28
Mild/Moderate	42.67	±21.82	36.29	24.12 - 60.07
Severe	38.43	±21.12	36.02	23.04 - 56.42
B1	48.47	±19.57	47.94	32.67 - 62.31
B2	37.46	±19.67	32.52	23.07 - 53.35
B3	25.01	±15.06	22.96	12.45 - 38.96
Terminal Ileum	53.08	±24.14	52.09	30.88 - 76.11
Ileocolonic	37.7	±17.28	35.5	24.87 - 50.94
Colonic	45.3	±20.43	47.94	26.67 – 62.63
Undefined	27.54	±2.65	26.77	25.35 – 30.49
UC	**45.07**	**±17.84**	**42.56**	**30.83 - 60.26**
Remission	45.79	13.78	45.89	32.71 - 60.94
Active	44.87	±19.06	42.01	29.10 - 61.08
18-40 years	45.22	±18.68	43.57	27.37 - 58.22
>40 years	41.24	±16.02	37.92	30.03 - 48.81
Mild/Moderate	41.98	±19.47	36.59	26.34 - 52.96
Severe	48.55	±18.78	43.28	31.92 - 63.94
Left-sided	43.19	±16.17	40.11	29.69 - 59.65
Pancolitis	47.33	±17.79	44.5	32.68 - 58.13
Undefined	48.42	±42.29	48.42	18.51 - 78.32

**Fig. 4:**
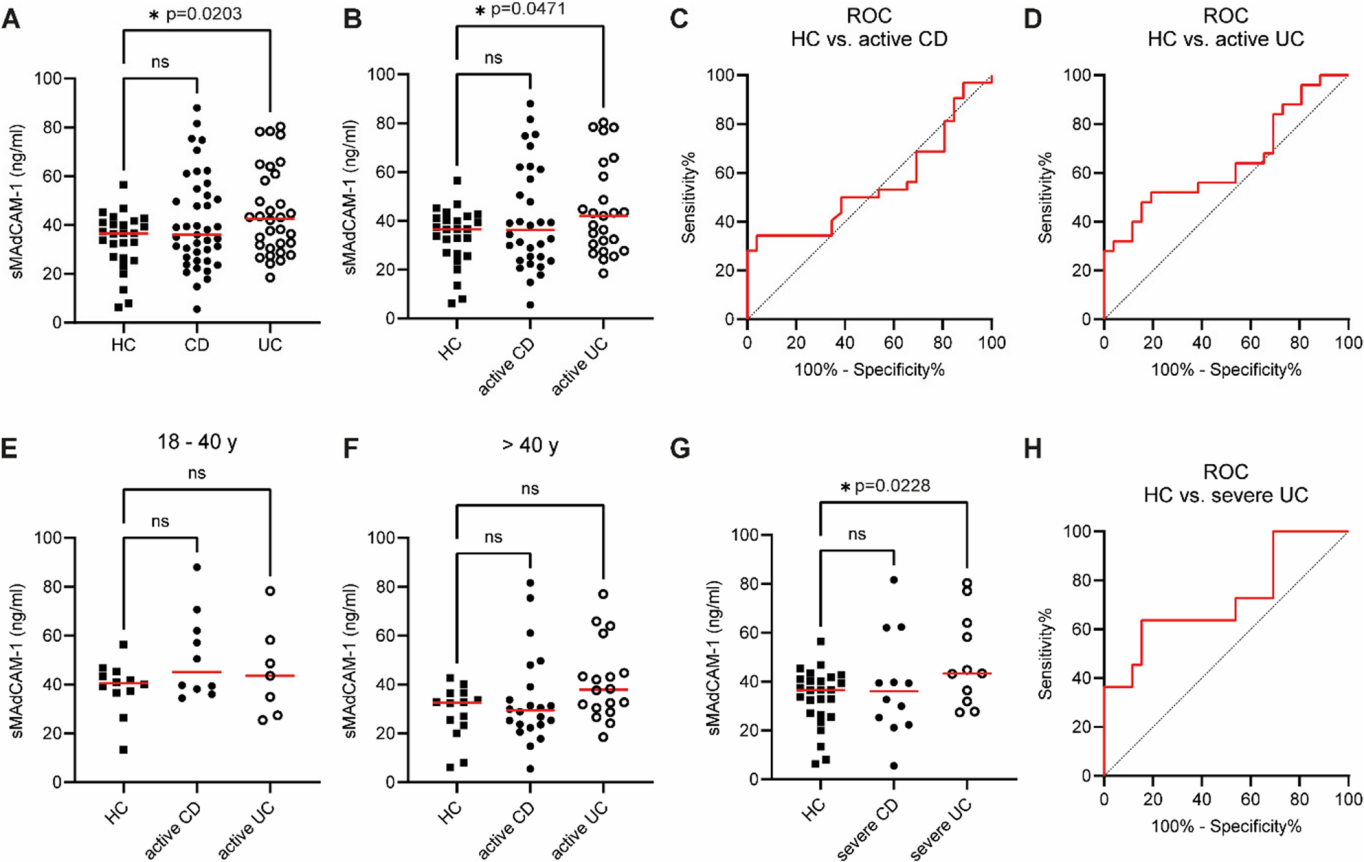
sMAdCAM-1 levels are increased in severe ulcerative colitis. sMAdCAM-1 concentrations were determined by sMAdCAM-1 detection assay in serum samples from 26 adult healthy individuals without intestinal inflammation and adult patients with CD (*n* = 41) or UC (*n* = 32) (A). IBD patients were categorized into remission or active disease (B) and further classified as having a severe course of disease based on their calprotectin levels (G). sMAdCAM-1 levels in adult UC patients differed significantly from healthy controls (A, B) and were particularly elevated in patients with severe UC (G), whereas no differences were observed between healthy controls and CD patients (A, B, G). Receiver operating characteristic (ROC) curves indicated that sMAdCAM-1 has no diagnostic utility for active CD (C) or active UC (D), but the assay demonstrated limited discrimination between healthy controls and UC patients with a severe course of disease (H). Significant differences were not observed when analyses were performed within age-stratified subpopulations (E, F). Dots represent individual samples and p values of Tukey post-hoc tests are indicated. Red horizontal lines represent the mean

sMAdCAM-1 concentrations were significantly increased in adult UC patients (45.07 ± 17.84 ng/ml) compared with healthy controls (33.30 ± 11.92 ng/ml; *p = 0.0203), whereas sMAdCAM-1 levels in adult CD patients (41.03 ± 19.32 ng/ml) did not differ significantly from either UC patients or healthy controls (Fig. 4A). This observation was also evident when sMAdCAM-1 levels were compared between UC (44.87 ± 19.06; *p = 0.0471) and CD (41.08 ± 21.32) patients with active disease and healthy controls (Fig. 4B). To evaluate a potential diagnostic significance of sMAdCAM-1 levels in adult IBD, we plotted ROC curves and calculated the AUC for both CD and UC patients compared to healthy controls. The sMAdCAM-1 bioassay failed to discriminate active CD from healthy controls (AUC = 0.55, *p* = 0.516) (Fig. 4C) or reliably distinguish active UC from healthy controls (AUC = 0.64, *p* = 0.07) (Fig. 4D).

Given the age-related decline in sMAdCAM-1 levels observed in Figs. 2 and 3, we subdivided healthy controls, as well as active CD and UC patients, into two age groups (< 40 years and ≥ 40 years) to assess an age-related bias. No significant differences in sMAdCAM-1 concentrations were observed in either of the sub-analyses between these groups (active CD (18-40y): 51.59 ± 17.76 ng/ml, active UC (18-40y): 45.22 ± 18.68 ng/ml; active CD (> 40y): 33.91 ± 18.79 ng/ml, active UC (> 40y) 41.24 ± 16.02 ng/ml) (Fig. 4E and F; Table 3). Specifically, the previously noted elevation of sMAdCAM-1 levels in active UC was no longer evident when the data were stratified by age.

Next, we compared sMAdCAM-1 concentrations across clinical subgroups of CD and UC patients. The localization of the inflammation did not influence sMAdCAM-1 levels in both CD and UC patients (data not shown, Table 3). Lower sMAdCAM-1 concentrations in serum samples of CD patients were associated with a penetrating behavior (B3) of disease (25.01 ± 15.06 ng/ml) and differed significantly from values obtained from serum samples of patients with non-stricturing/non-penetrating (B1) CD (48.47 ± 19.57 ng/ml; *p = 0.018) (data not shown, Table 3). However, this difference may be partially confounded by the older age of CD patients with penetrating disease (median age: 46.7 years; IQR_25−75_ 31.5–55.4) compared to those with non-stricturing/non-penetrating disease (median age: 40.1 years; IQR_25−75_ 27.2–48.1).

Finally, we analyzed sMAdCAM-1 concentrations in CD and UC patients with a severe course of disease (fecal calprotectin > 500 mg/kg). Significantly elevated sMAdCAM-1 levels were observed only in patients with severe UC (*n* = 11; 48.55 ± 18.78 ng/ml; *p = 0.0228), whereas severe CD patients (*n* = 12) showed no significant change in sMAdCAM-1 levels (38.43 ± 21.12 ng/ml) (Fig. 4G). ROC curve analysis for the detection of severe UC using sMAdCAM-1 levels (AUC = 0.72; *p = 0.03) demonstrated that, at a cut-off of > 35.06 ng/ml, the assay achieved a sensitivity of 72.7% and a specificity of 46.2% (Fig. 4H).

### Detection of pediatric ulcerative colitis through increased levels of sMAdCAM-1.

We collected serum samples from pediatric CD (*n* = 17) and UC (*n* = 15) patients. Characteristics of both IBD entities are presented in Table 2. Median ages of pediatric CD (17.1 years; IQR_25−75_ 14.4–17.6) and pediatric UC patients (13.8 years; IQR_25−75_ 10.4–15.7) differed slightly, but all pediatric IBD patients (100%) were older than 8 years. In the pediatric CD patients 47.1% (*n* = 8) were currently in remission while 52.9% (*n* = 9) suffered from active CD (severe CD: *n* = 6; 35.3%). Only one pediatric UC patient (6.7%) was in clinical remission while the rest (*n* = 14; 93.3%) exhibited symptoms of colitis (severe UC: *n* = 7; 46.7%). 5 pediatric IBD patients underwent off-label vedolizumab treatment at the time-point of sample acquisition (Table 2). A summary of sMAdCAM-1 concentration data for all cohorts is provided in Table 4.

**Table 4:** Serum sMAdCAM-1 levels (ng/ml) of pediatric cohorts (healthy controls and IBD patients)

	**Mean**	**SD**	**Median**	**IQR**_25-75_
Healthy controls	**62.35**	**±25.36**	**60.14**	**45.02 - 72.43**
0-8 years	79.7	±26.65	67.05	62.06 - 94.75
8-18 years	50.69	±16.35	48.86	36.04 - 65.93
8-14 years	56.24	±12.42	56.41	47.26 - 66.74
14-18 years	46.25	±18.32	38.65	33.15 - 62.38
Pediatric IBD patients	**49.45**	**±22.72**	**44.55**	**30.67 - 71.09**
8-14 years	62.19	±22.83	68.02	41.01 - 82.71
14-18 years	43.08	±20.37	39.72	26.01 - 63.08
CD	**33.12**	**±12.69**	**37.35**	**19.54 - 44.41**
Remission	33.68	±13.35	39.59	17.82 - 44.55
Active	32.62	±12.98	32.76	20.45 - 39.25
8-14 years	30.32	±10.10	30.32	23.18 - 37.46
>14 years	33.39	±14.59	32.76	19.24 - 44.15
Mild/Moderate	38.6	±1.76	38.6	37.35 - 39.84
Severe	30.63	±14.71	25.68	19.24 - 42.37
Terminal Ileum	29.47	±24.01	17.82	13.51 - 57.08
Ileocolonic	27.1	±10.15	25.11	18.64 - 37.55
Colonic	37.49	±8.05	38.53	30.47 - 44.52
Azathioprine	36.43	±13.87	35.13	25.58 - 48.19
Azathioprine + Corticosteroid	23.18	±0.00	23.18	
TNFa antagonist	39.79	±4.13	37.46	37.35 - 44.55
TNFa antagonist + Azathioprine	27.13	±13.97	19.54	15.93 - 42.13
a4b7 antagonist + Azathioprine	<0.41	±0.00	<0.41	
UC	**69.87**	**±14.00**	**71.28**	**63.08 - 79.44**
Remission	35.33	±0.00	35.33	
Active	73.01	±9.24	71.46	66.93 - 80.14
8-14 years	75.76	±11.14	76.39	65.88 - 86.21
>14 years	69.72	±5.78	71.09	64.36 - 74.40
Mild/Moderate	71.93	±9.95	70.33	63.35 - 82.12
Severe	73.63	±9.57	71.46	66.93 - 80.14
Azathioprine	63.17	±16.89	71.09	47.40 - 74.98
TNFa antagonist + Azathioprine	74.66	±10.23	71.46	66.93 - 85.28
a4b7 antagonist + Azathioprine	<0.41	±0.00	<0.41	

**Fig. 5:**
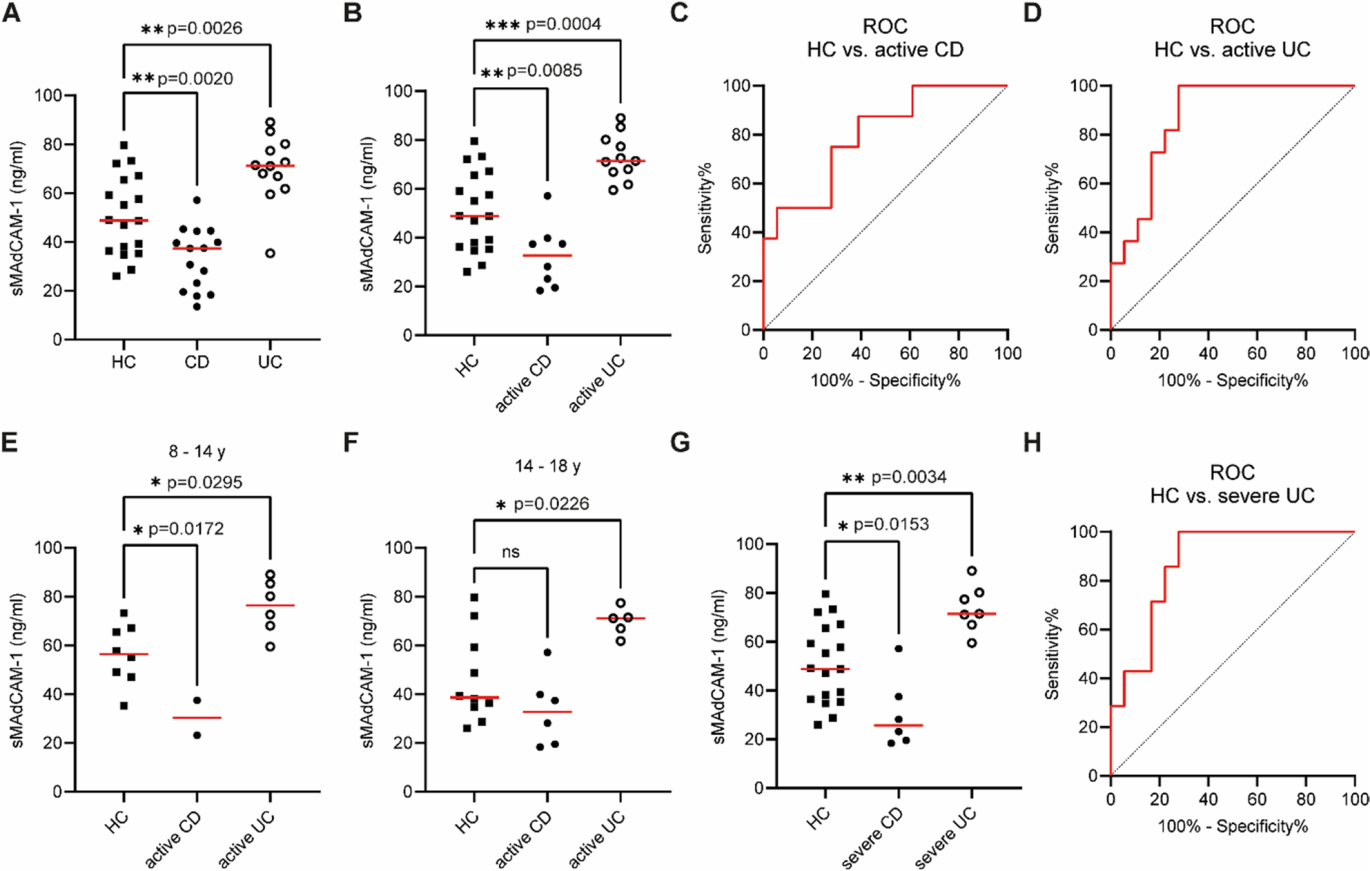
Different levels of sMAdCAM-1 discriminate pediatric ulcerative colitis from healthy controls. sMAdCAM-1 concentrations in serum samples from 18 pediatric individuals (HC, age >8 years) and 32 pediatric IBD patients (17 CD and 15 UC patients) were determined by sMAdCAM-1 detection assay. sMAdCAM-1 levels in pediatric HC, CD and UC differed significantly (**A**). Patients were categorized as under remission or with active disease (**B**) and further classified as having a severe course of disease based on their calprotectin levels (**G**). Pediatric patients with active and severe pediatric CD exhibited markedly decreased sMAdCAM-1 levels, whereas active and severe UC patients showed significantly elevated sMAdCAM-1 levels (**B**, **G**). Receiver operating characteristic (ROC) curves demonstrated that the sMAdCAM-1 bioassay could detect active CD (**C**), active UC (**D**), and, most notably, severe UC (**H**). Increased sMAdCAM-1 levels in active pediatric UC were consistently observed in age-stratified subpopulations (**E**, **F**). Dots represent individual samples and p values of Tukey post-hoc tests are indicated. Red horizontal lines represent the mean

First, we compared serum levels of sMAdCAM-1 from healthy children older than 8 years (*n* = 18; median age 14.1 years; IQR_25−75_ 11.7–15.7) with serum levels from pediatric IBD patients. sMAdCAM-1 concentrations from serum of pediatric IBD patients differed clearly from levels detected in age-matched healthy controls (50.69 ± 16.35 ng/ml) (Fig. 5A). Pediatric UC was characterized by a marked elevation of sMAdCAM-1 levels (69.87 ± 14.00 ng/ml, **p = 0.0026) compared to healthy controls. Furthermore, we detected significantly decreased sMAdCAM-1 concentrations in serum samples of CD patients (33.12 ± 12.69 ng/ml) in comparison to serum samples of healthy subjects (**p = 0.0020).

This pattern was reflected in patients with active disease, with markedly elevated sMAdCAM-1 levels in active UC (73.01 ± 9.24 ng/ml; ***p = 0.0004) and comparatively lower levels in active CD (32.62 ± 12.98 ng/ml; **p = 0.0085) (Fig. 5B). ROC curve analysis for the detection of active CD using sMAdCAM-1 levels (AUC = 0.80; *p = 0.017) demonstrated that, at a cut-off of < 43.4 ng/ml, the assay achieved a sensitivity of 87.5% and a specificity of 61.1% (Fig. 5C). In contrast, sMAdCAM-1 levels > 59.3 ng/ml identified active UC with a sensitivity of 100% and a specificity of 72.2% (AUC = 0.87; **p = 0.001) (Fig. 5D).

Although the median ages of healthy controls and UC patients were well matched, an age-related decline in sMAdCAM-1 concentrations during adolescence – observed in both healthy controls (Figs. 2D, E) and IBD patients (Figs. 3D, E) – could have influenced our results. We therefore once again subdivided all three groups (healthy controls, active CD and active UC) into two subgroups (8–14 years and 14–18 years). Even in these sub-analyses, active UC was associated with significantly elevated sMAdCAM-1 levels (8–14 years: 75.76 ± 11.14 ng/ml; *p = 0.0295; 14–18 years: 69.72 ± 5.78 ng/ml; *p = 0.0226), whereas active CD patients showed markedly decreased sMAdCAM-1 concentrations (8–14 years: 30.32 ± 10.10 ng/ml; *p = 0.0172; 14–18 years: 33.3 ± 14.59 ng/ml) (Figs. 5E, F).

We assessed sMAdCAM-1 levels across clinical subgroups of our pediatric IBD cohorts. In the pediatric CD cohort, the highest sMAdCAM-1 concentrations were observed in patients with colonic inflammation (37.49 ± 8.05 ng/ml), whereas those with terminal ileitis (29.47 ± 24.01 ng/ml) or ileocolonic localization (27.10 ± 10.15 ng/ml) exhibited comparably low sMAdCAM-1 levels. Interestingly, all IBD patients undergoing off-label vedolizumab treatment displayed undetectable serum levels of sMAdCAM-1 (< 0.41 ng/ml) (Table 4, data not shown).

Finally, we measured sMAdCAM-1 levels in clinical subgroups of pediatric IBD patients with a severe course of disease. sMAdCAM-1 levels were markedly elevated in pediatric patients with severe UC (*n* = 7; 73.63 ± 9.57 ng/ml; **p = 0.0034) whereas sMAdCAM-1 levels were significantly decreased in pediatric patients with severe CD (*n* = 6; (30.63 ± 14.71 ng/ml; *p = 0.0153) (Fig. 5G). Furthermore, ROC curve analysis for the detection of severe UC using sMAdCAM-1 levels (AUC = 0.87; **p = 0.0044) demonstrated that, at a cut-off of > 66.23 ng/ml, the assay achieved a sensitivity of 85.7% and a specificity of 77.8% (Fig. 5H).

## Discussion

Our single-center study was, to our knowledge, the first to investigate the role of sMAdCAM-1 as a potential biomarker for IBD in both pediatric and adult patients. We identified three key findings that reflect the relevance of MAdCAM-1 and its soluble form for (1) gut maturation, (2) IBD pathophysiology, and (3) as a potential diagnostic tool in pediatric UC. First, we observed a non-linear decline in sMAdCAM-1 concentrations across childhood, adolescence, and adulthood (Figs. 2 and 3). Second, sMAdCAM-1 shedding was increased in patients with UC, specifically in adults with severe disease and in pediatric patients with active UC (Figs. 4 and 5). Third, our data support the utility of sMAdCAM-1 as a potential biomarker for pediatric UC (Fig. 5).

Leung et al. first described in 2004 a bioassay detecting soluble MAdCAM-1in body fluids, i.e., serum and urine of healthy donors 29. Pro-inflammatory TNF-α upregulates MAdCAM-1 gene expression 8 and enhanced MAdCAM-1 tissue expression hallmarks inflamed mucosal sites in human IBD [2, 25]. Circulating cell adhesion molecules (soluble CAMs) shed from endothelial cells at inflamed sites can serve as serologic biomarkers in acute and chronic disease [26, 27]. Consequently, the sMAdCAM-1 bioassay presents a potential diagnostic approach with respect to IBD [30–33].

In this context, sMAdCAM-1 bioassays were recently performed to evaluate treatment effects in patients with IBD receiving vedolizumab or ontamalimab, a monoclonal antibody targeting MAdCAM-1 that has been developed for the treatment of CD and UC. Decreased sMAdCAM-1 levels were observed in CD patients during active treatment with ontamalimab across different dosage groups, whereas placebo administration did not affect serum sMAdCAM-1 levels 30. In addition, median plasma sMAdCAM-1 levels significantly decreased during vedolizumab therapy in patients with CD [31] and UC. However, the sMAdCAM-1 bioassay failed to reliably predict clinical and endoscopic remission in these patients [32, 33]. In conclusion, the sMAdCAM-1 bioassay appears capable of monitoring pharmacodynamics in vedolizumab treated IBD patients, and insufficient vedolizumab treatment is associated with increasing sMAdCAM-1 plasma levels. From our pediatric cohort, all 5 IBD patients under vedolizumab therapy exhibited reduced sMAdCAM-1 levels that were below the detection limit (Table 4). We speculate that blocking of α4β7/MAdCAM-1 interactions indirectly downregulates MAdCAM-1 expression on HEV in IBD patients, leading to a detectable reduction of sMAdCAM-1 shedding. If so, α4β7/MAdCAM-1 mediated lymphocyte/endothelial interaction may be a crucial step perpetuating the ongoing expression of MAdCAM-1 in inflamed gut tissues. In this regard, we recently showed that β7 integrin expression on lymphocytes contributes to MAdCAM-1 upregulation in the mouse model of concanavalin A (ConA)-induced hepatitis 34.

We determined increased sMAdCAM-1 levels in adult patients suffering from active (Fig. 4B) and, most notably, severe UC (Fig. 4G). In contrast, CD patients exhibit no elevation of sMAdCAM-1 levels either in active or in severe disease (Figs. 4B, G). This was mirrored by our findings in the pediatric cohort. The sMAdCAM-1 expression levels of pediatric UC patients clearly increased, whereas pediatric CD patients exhibited significantly decreased sMAdCAM-1 expression (Figs. 5B, G). Receiver operating characteristic (ROC) curves demonstrated that the sMAdCAM-1 bioassay can detect active CD, active UC, and particularly severe UC in pediatric patients (Figs. 5C, D, H), but cannot reliably distinguish healthy adults from adult UC patients with active or severe disease (Figs. 4D, H). Nevertheless, age-related effects on sMAdCAM-1 levels were apparent across all cohorts, and the observed differences in the adult cohort largely disappeared (Figs. 4E, F) or were mitigated in the pediatric cohort (Figs. 5E, F) when analyses were restricted to age-matched subgroups.

Our findings may, however, still inform future therapeutic considerations, either by enabling a more precise distinction between the two major forms of IBD or by supporting the prognostic identification of UC patients with severe disease activity who may require early biological treatment.

Moreover, these results suggest a more predominant function of MAdCAM-1 in UC, whereas MAdCAM-1 function in CD appears secondary. In this regard, it is interesting to note that the monoclonal anti-MAdCAM-1 antibody ontamalimab sufficiently induced remission in active UC [35–37], but failed to improve active CD [38, 39]. Recently, Keir et al. described the upregulation of gene expression levels for different adhesion molecules in colon and ileum of IBD patients. *MAdCAM-1* gene expression was significantly upregulated in colonic tissues of UC patients, especially at inflamed sites. Uninflamed and inflamed colon and ileum tissues from CD patients also showed enhanced *MAdCAM-1* gene expression. Yet, the variability of *MAdCAM-1* gene expression was comparably large in CD patients. Moreover, gene expression of *VCAM-1* at uninflamed or inflamed colonic tissues was significantly elevated in CD but not UC patients 2. We postulate that decreased sMAdCAM-1 levels in pediatric and adult CD patients may result from reduced MAdCAM-1 expression on HEV, pointing to alternative pathways (ICAM-1, VCAM-1) of leukocyte migration in this situation.

Finally, our data showed a change in the expression of sMAdCAM-1 during childhood, adolescence, and adulthood (Figs. 2 and 3). This reduction in sMAdCAM-1 levels is consistent with findings from other studies on MAdCAM-1 and α4 integrin both in humans and animal models. Correspondingly, the density of MAdCAM-1 expression in the intestinal lamina propria is diminished in older compared to younger rats [40, 41]. Moreover, decreased intestinal CD4 T cell trafficking has been observed for elderly humans 42, and α4 integrin expression on circulating T cells is significantly lower in older (mean age 71.2 ± 0.52) compared to younger humans (mean age 22.7 ± 0.73) 43. Age-related immunosenescence generally leads to a less severe course of IBD, but increased susceptibility to infections in elderly patients 44.

In contrast to the situation in adulthood and senescence, at the beginning of life embryonic MAdCAM-1 is widely expressed in different tissues and gradually becomes polarized to mucosal HEV after birth in humans. In infancy, mucosal sites are massively colonized by commensal microbiota and are exposed for the first time to environmental factors like food antigens. Both could represent a strong stimulus for increased intestinal MAdCAM-1 expression during childhood. This assumption is reflected by the opposing findings of decreased ileal MAdCAM-1 expression in mice and patients treated by broad-spectrum antibiotics. Moreover, patients treated with antibiotics displayed significantly decreased sMAdCAM-1 serum levels compared with controls 45. These findings, together with our results, underscore how serum detection of sMAdCAM-1 could reflect intestinal MAdCAM-1 expression under different conditions (i.e., homeostasis, bacterial colonization, or inflammation).

## Conclusions

The results of our study on serum levels of sMAdCAM-1 in children and adults reflect the critical relevance of MAdCAM-1 for gut homeostasis and inflammation and point to the importance of MAdCAM-1 for the maturation of the intestinal immune system. Pediatric and adult severe UC are accompanied by augmented MAdCAM-1 shedding. Therefore, measurement of increased sMAdCAM-1 levels in serum could serve as a potential biomarker for UC. However, these findings are based on relatively small patient cohorts and lack longitudinal follow-up. Another limitation of our study is the absence of newly diagnosed, treatment-naïve IBD patients. Future studies, including larger cohorts and repeated measurements across different disease states (at diagnosis, during remission, and at flare), are therefore required to substantiate these conclusions. 

## Data Availability

The datasets generated during this study are available from Figshare data repository with the following URL: https://doi.org/10.6084/m9.figshare.19803835.v1](file:/C:/Users/mmuschaweck/Desktop/The%20datasets%20generated%20during%20this%20study%20are%20available%20from%20Figshare%20data%20repository%20with%20the%20following%20URL:%20https:/doi.org/10.6084/m9.figshare.19803835.v1) and upon request from corresponding author
